# Clinical role of miR-421 as a novel biomarker in diagnosis of gastric cancer patients: A meta-analysis

**DOI:** 10.1097/MD.0000000000029242

**Published:** 2022-05-20

**Authors:** Yingying Xu, Guiping Wang, Wenqing Hu, Songbing He, Dandan Li, Ping Chen, Jinjie Zhang, Yongshun Gao, Duonan Yu, Liang Zong

**Affiliations:** a Department of General Surgery, Yizheng People's Hospital, Clinical Medical College, Yangzhou University, Yangzhou, Jiangsu Province, China; b Department of Gastrointestinal Surgery, Clinical Medical School of Yangzhou University, Northern Jiangsu People's Hospital, Yangzhou, Jiangsu, PR China; c Clinical Medical College, Dalian Medical University, Liaoning, PR China; d Department of Gastrointestinal Surgery, Changzhi People's Hospital, The Affiliated Hospital of Shanxi Medical University, Changzhi, Shanxi, PR China; e Department of General Surgery, The First Affiliated Hospital of Soochow University, Suzhou, PR China; f Department of Gastrointestinal Surgery, The Affiliated Heji Hospital of Changzhi Medical college, Changzhi, Shanxi, PR China; g Department of Gastrointestinal Surgery, The First Affiliated Hospital of Zhengzhou University, Zhengzhou, Henan, PR China; h Jiangsu Key Laboratory of Experimental & Translational Non-coding RNA Research, Yangzhou University School of Medicine, Yangzhou, PR China.

**Keywords:** diagnostic value, gastric cancer, meta-analysis, microRNA

## Abstract

**Background:**

Gastric cancer (GC) has been identified as one of the most common malignancies. It was found that microRNAs can be used as potential biomarkers for GC diagnosis. The aim of this study was to estimate the diagnostic value of 4 potential microRNAs in GC.

**Methods:**

PubMed, Embase, Cochrane Library, and Web of Science were used to search published studies. The quality of the studies was scored with the Quality Assessment of Diagnostic Accuracy Studies. The pooled sensitivity and specificity, diagnostic odds ratio (DOR) and area under the curve (AUC) were calculated. The heterogeneity was evaluated using Cochrane Q statistics and the inconsistency index.

**Results:**

A total of 22 studies reporting the diagnostic value of miR-21 (n = 9), miR-106 (n = 10), miR-421 (n = 5) and miR-223 (n = 3) were included. Quality Assessment of Diagnostic Accuracy Studies scores showed the high quality of the selected 22 articles. The random effects model was adopted by evaluating the heterogeneity between articles. The DOR, AUC, and Q value of miRNA-21 were 12.37 (95% confidence interval [CI]: 5.36–28.54), 0.86 and 0.79, respectively. The DOR, AUC and Q value of miRNA-106 were 12.98 [95% CI: 7.14–23.61], 0.85 and 0.78, respectively. The DOR, AUC and Q value of miRNA-421 were 27.86 [95% CI: 6.04–128.48], 0.92 and 0.86, respectively. The DOR, AUC and Q value of miRNA-223 were 18.50 [95% CI: 7.80–43.86], 0.87 and 0.80, respectively. These results indicate that miRNA-421 has the highest diagnostic accuracy, followed by miR-223, miRNA-21, and miRNA-106 among the 4 microRNAs in GC.

**Conclusions:**

miR-21, miR-106, miR-421, and miR-223 have good diagnostic efficacy, especially miR-421, could be used as auxiliary diagnostic indicator for GC.

## 1 Introduction

Gastric cancer (GC) is one of the most common gastrointestinal malignant tumors that seriously harm human health. According to the latest statistics, GC is a leading cancer worldwide and is responsible for over 1,000,000 new cases and an estimated 769,000 deaths in 2020, making it the fifth most frequently diagnosed cancer and the third leading cause of cancer death.^[1]^ In spite of the incidence and mortality of both males and females showing a downward trend,^[2]^ it could be concluded that the incidence of early GC is consistently increasing.^[3,4]^ Therefore, accurate early diagnosis of GC is critical for early treatment and improved prognosis of GC patients. GC is commonly diagnosed by gastroscopy, surgical biopsy, and some noninvasive methods such as evolutionary endoscopy and positron emission tomography.^[5]^ However, due to the invasiveness or high cost, these methods have not been widely used in the early diagnosis of GC. Thus, it is necessary to find biomarkers for the early diagnosis of GC. Traditional tumor biomarkers for GC, including cancer embryo antigen, pepsinogen, carbohydrate antigen 199, carbohydrate antigen 724 and gastrin-17, have been applied in clinical practice, but with insufficient sensitivity and specificity.^[6,7]^ It is of great practical significance to search for suitable diagnostic markers for mass screening of GC.

MicroRNA is a class of short non-protein-coding RNAs with a length of 18 to 25 nucleotides that have been implicated in the regulation of gene post-transcriptional modification and almost all signaling pathways in cells.^[8,9]^ Numerous studies demonstrate that microRNAs are dysregulated in various tumors and have a causal relationship with cell cycle, apoptosis and migration which depicts their potential as effective biomarkers of tumor diagnostic and prognostic.^[10,11]^ They are stable in plasma or serum and are readily available, which is attractive for researchers. By observing the expression profile of microRNAs in different digestive cancer, it was found that microRNAs can be used as potential biomarkers for tumor diagnosis.^[10]^ Thus, it is promising to explore the diagnostic value of microRNAs for GC.

At present, many microRNAs and their targets have been found to be closely related to the proliferation, invasion, metastasis and apoptosis of GC cells and the treatment and prognosis of GC. Studies have shown that miR-21 regulates the occurrence and development of various cancers, such as non-small cell lung cancer, GC, colorectal cancer and ovarian cancer.^[12–15]^ Exosome miR-21–5P promotes peritoneal metastasis of GC through mesothelial-to-mesenchymal transition,^[16]^ so miR-21 can be used as a potential biomarker for predicting peritoneal recurrence of GC.^[17]^ MiR-106 belongs to the miR-17 family, one of the most common studied onco-miRNA groups. In vivo and in vitro experiments showed that miR-106 promoted metastasis of early GC by targeting ALEX1. Comprehensive analysis identified miR-106 as a molecular marker for GC.^[18]^ At present, the related studies of miR-421 mainly focus on gastrointestinal carcinomas and genital carcinomas. In gastrointestinal cancers, such as gastric cancer, esophageal cancer, colorectal cancer, biliary cancer and liver cancer, miR-421 acts as a carcinogen miRNA to promote cancer development.^[19–21]^ In biliary tract cancer and liver cancer, miR-421 promoted cell proliferation and migration by down-regulating farnesoid X receptor.^[22,23]^ The gene encoding miR-223 is located at q12 site of X chromosome, and miR-223 play a regulatory role as both tumor promoter and tumor suppressor. MiR-223 regulates cell differentiation, proliferation, apoptosis and metastasis as a tumor suppressor in leukemia, lymphoma, oral cancer, lung cancer and breast cancer.^[24]^ MiR-223 was up-regulated in human gastric cancer tissue samples, FBXW7/hCdc4 (FBW7)^[25]^ and RhoB^[26]^ and Stathmin1^[27]^ as the target genes of miR-223 regulate the occurrence and development of GC and drug resistance. Macrophage-derived miR-223 transfer leads to adriamycin resistance in GC.^[28]^ Therefore, we selected miR-21, miR-106, miR-421, and miR-223 upregulated in GC and compared the diagnostic value of these 4 microRNAs in GC through meta-analysis.

## 2 Materials and methods

### 2.1 Search strategy

Keywords including

1. (gastric cancer [All Fields] OR gastric carcinoma [All Fields] OR stomach cancer [All Fields] OR stomach carcinoma [All Fields]),
2. (microRNA-21 [All Fields] OR miR-21 [All Fields] OR miRNA-21[All Fields] OR hsa-miR-21[All Fields]),
3. (microRNA-106 [All Fields] OR miR-106 [All Fields] OR miRNA-106 [All Fields] OR hsa-miR-106 [All Fields]),
4. (microRNA-421 [All Fields] OR miR-421 [All Fields] OR miRNA-421[All Fields] OR hsa-miR-421 [All Fields]),
5. (microRNA-223 [All Fields] OR miR-223 [All Fields] OR miRNA-223 [All Fields] OR hsa-miR-223 [All Fields]) were searched on PubMed, Embase, Cochrane Library and Web of Science up to May of 2021.

**Figure 1:**
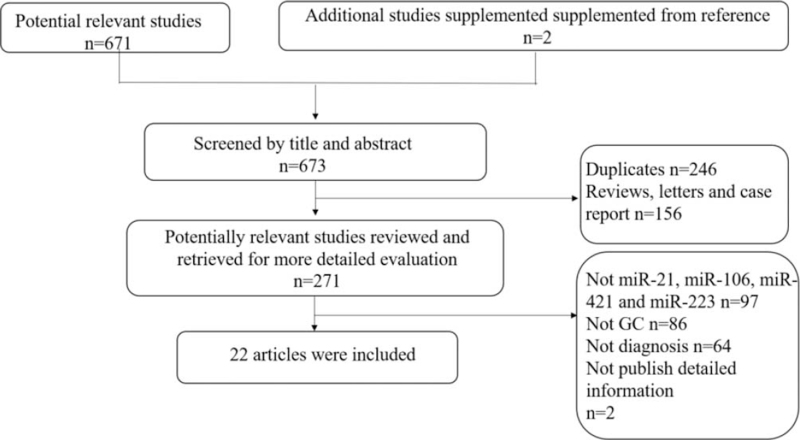
Flowchart of literature selection.

The search strategy was (1) and (2), (1) and (3), (1) and (4), and (1) and (5). The language was limited to English, and the subject was limited to humans. We also searched the articles of reference to obtain additional studies. Finally, all literature identified according to the search strategy was independently evaluated by 2 researchers. If there was any disagreement, discussion was conducted, or a third researcher was consulted for a consensus. The search strategies are depicted in Figure 1.

### 2.2 Inclusion and exclusion criteria

The inclusion criteria were as follows:

1. the articles were published in English and full text was available;
2. the diagnosis of GC was made by histopathology;
3. the sample types included plasma, serum, blood and others;
4. patients with benign diseases or healthy people were selected as the control group; and
5. the studies had sensitivity, specificity or other data to calculate true positive, false positive, false negative and true negative.

The exclusion criteria were as follows:

1. unqualified data;
2. duplicate publications;
3. non-experimental studies, such as case reports, reviews and letters; and
4. no full text.

### 2.3 Study selection and data extraction

The screening was in strict accordance with the inclusion and exclusion criteria. Data for each study were retrieved independently by 2 reviewers and divergences were resolved by discussing with the third researchist. Characteristics of the studies included first author, publication year and country, and characteristics of the subjects included number of patients, age, sample type, pathologic stage and detection methods. According to the numbers of experimental and control groups, sensitivity and specificity, we calculated the true positive, false positive, false negative and true negative. The main characteristics of the included studies are presented in Table 1.

**Table 1:** The main characteristics of the included studies.

First author	Country	Patients (Controls)	Mean or median age	Stage I, II%	Sample type	Detection methods	TP	FN	FP	TN
**microRNA-21**										
Cui L	China	42 (99)	64.2	NR	Gastric juice	qRT-PCR	36	6	2	97
Li BS	China	70 (70)	54	33	Plasma	qRT-PCR	52	18	17	53
Wu J	China	50 (50)	NR	40	Serum	qRT-PCR	44	6	10	40
Liu HN	China	80 (82)	65.1	47.5	Serum	qRT-PCR	62	18	47	35
Zheng Y	China	53 (20)	60	30.2	Blood	qRT-PCR	44	9	4	16
Wang B	China	30 (39)	58	36.7	Serum	qRT-PCR	17	13	2	37
Shiotani A	Japan	62 (70)	67.8	100	Serum	qRT-PCR	36	26	10	60
Tsujiura M	Japan	69 (30)	NR	73.9	Plasma	qRT-PCR	42	27	12	18
Shen J	China	29 (25)	54	NR	Serum	qRT-PCR	15	14	2	23
**microRNA-106**										
Zhou H	China	90 (27)	61.4	NR	Serum	qRT-PCR	43	47	3	24
Zeng Q	China	40 (36)	NR	22.5	Serum	qRT-PCR	30	10	3	33
Li F	China	65 (65)	54.1	40	Plasma	qRT-PCR	56	9	5	60
Hou X	China	80 (60)	68	56.3	Plasma	qRT-PCR	62	18	4	56
Tsujiura M	Japan	69 (30)	NR	73.9	Plasma	qRT-PCR	55	14	11	19
Cai H	China	90 (90)	46.2	38.9	Plasma	qRT-PCR	59	31	18	72
Shiotani A	Japan	62 (70)	67.8	100	Serum	qRT-PCR	47	15	34	36
Cui L	China	42 (99)	64.2	NR	Gastric juice	qRT-PCR	31	11	11	88
Wang N	China	110 (110)	NR	57.2	Serum	qRT-PCR	69	41	13	97
Yuan R	China	48 (22)	64	NR	Plasma	qRT-PCR	37	11	8	14
**microRNA-421**										
Zhou H	China	40 (17)	64.9	52.5	Mononuclear	qRT-PCR	38	2	6	11
Zhang X	China	42 (47)	56.8	NR	Gastric juice	qRT-PCR	30	12	13	34
Wu J	China	90 (90)	NR	58.9	Serum	qRT-PCR	81	9	13	77
Liu HN	China	80 (82)	65.1	47.5	Serum	qRT-PCR	75	5	65	17
Chen JL	China	90 (45)	NR	27.6	Plasma	qRT-PCR	87	3	2	43
**microRNA-223**										
Zhou XY	China	50 (50)	57.8	38	Plasma	qRT-PCR	35	15	10	40
Li BS	China	70 (70)	54	33	Plasma	qRT-PCR	59	11	8	62
Wang H	China	50 (47)	NR	62	Serum	qRT-PCR	41	9	10	37

TP, true positive; FN, false negative; FP, false positive; TN, true negative; NR, not report.

### 2.4 Quality assessment

**Figure 2:**
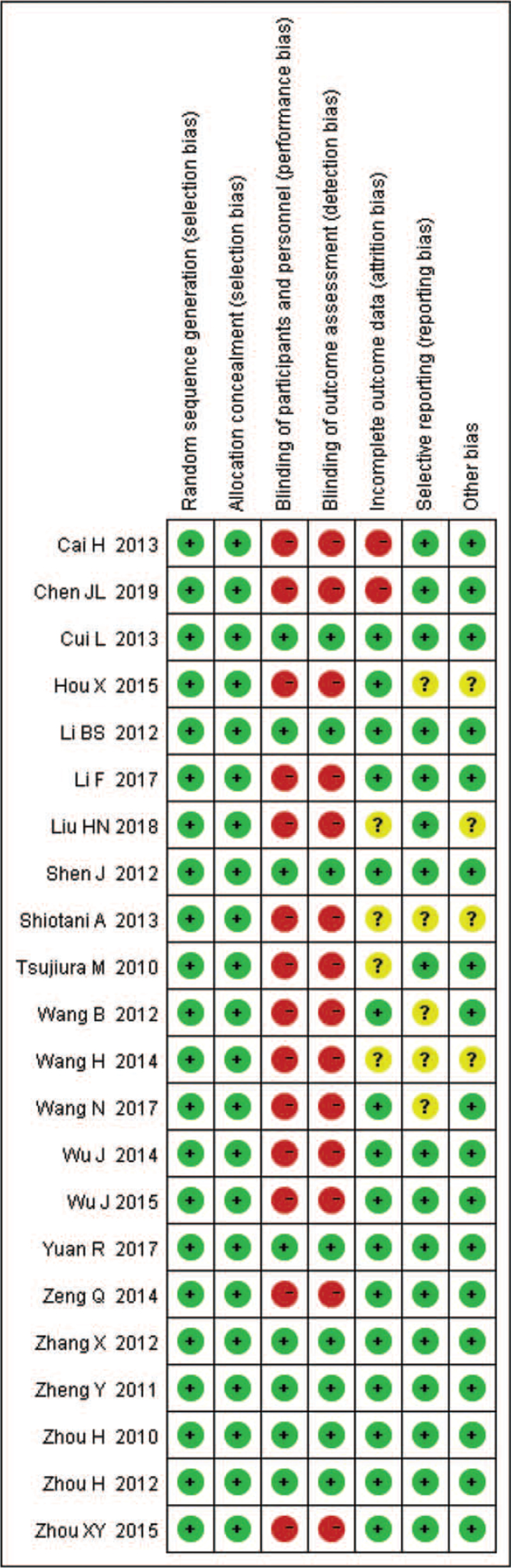
Risk of bias of each included study. Red cycle: study with high risk of bias. Green cycle: study with low risk of bias. Yellow cycle: study with insufficient information for assessing risk of bias.

Quality assessment was performed according to Quality Assessment of Diagnostic Accuracy Studies-2. Two researchers assessed the quality of studies separately, and any objections were resolved through discussion with the third investigator. The result is shown in Figure 2.

### 2.5 Statistical analysis

The data of the included studies were extracted and the diagnostic odds ratios (DOR) were combined according to the types of microRNA. The higher the DOR value, the better the diagnostic efficacy. The feasibility and accuracy of microRNA as diagnostic tools for GC was evaluated using receiver operating characteristic (ROC) curves and the area under the ROC curve (AUC). ROC curve was drawn according to sensitivity and specificity and the AUC of each microRNA was calculated respectively. The value of AUC ranged from 0.5 to 1.0, and the closer the value was to 1.0, the better the diagnostic accuracy was.

The Meta-Disc version 1.4 software package was used to perform statistical analysis. *P* value <.10 or *I*^2^ value >50% indicate high heterogeneity. If moderate or high heterogeneity was calculated, the random-effects model was utilized to pool the results. Otherwise, the fixed-effects model was used.

## 3 Results

### 3.1 Included studies

The initial search identified 673 articles among which 246 duplicates and 156 nonexperimental studies were excluded. The left 271 potentially relevant studies were reviewed and for more detailed evaluation. After intensive reading, 247 articles were excluded as not mentioned miR-21, miR-106, miR-421, and miR-223 (n = 97), diagnosis value (n = 64) and GC (n = 86), and additional 2 studies failed to publish detailed information (Fig. 1). Thus, a total of 22 full-text articles were included in this study.^[29–50]^

### 3.2 Study characteristics and quality assessment

In these studies, all the GC patients were diagnosed based on histopathology. The control individuals were all from healthy volunteers who had never been diagnosed with a malignant tumor. Among them, 9 articles reported the diagnostic value of microRNA-21, including 485 GC patients and 485 healthy controls.^[29–37]^ The sources of miR-21 were plasma (n = 2), serum (n = 5), gastric juice (n = 1), and blood (n = 1) in these studies. Ten articles reported the diagnostic value of microRNA-106, including 696 GC patients and 609 healthy controls.^[29,35,36,38–44]^ The sources of miR-106 were plasma (n = 5), serum (n = 4), gastric juice (n = 1) in these studies. Five studies reported the diagnostic value of microRNA-421, including 342 GC patients and 281 healthy controls.^[32,45–48]^ The sources of miR-421 were plasma (n = 1), serum (n = 2), gastric juice (n = 1), and mononuclear cells (n = 1) in these studies. And 3 studies reported the diagnostic value of microRNA-223, including 170 GC patients and 167 healthy controls.^[30,50,51]^ The sources of miR-223 were plasma (n = 2) and serum (n = 1) in these studies. All of the included studies were from China and Japan. Detection methods of microRNAs expression were mostly reverse transcription PCR (RT-PCR). The characteristics of each included study and of the patients are described in detail in Table 1. And Quality Assessment of Diagnostic Accuracy Studies-2 results were shown that no low-quality studies were included in this meta-analysis (Fig. 2).

### 3.3 Data analysis

**Figure 3:**
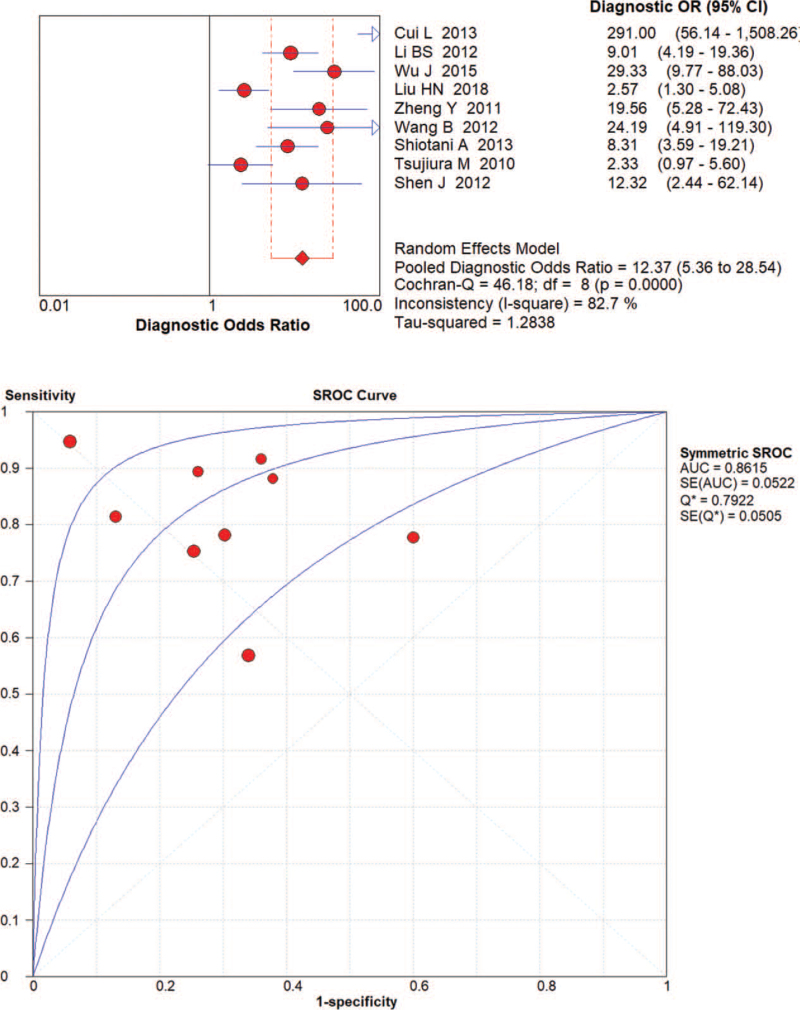
The DOR, AUC, and Q value of miR-21 in the diagnosis of GC.

**Figure 4:**
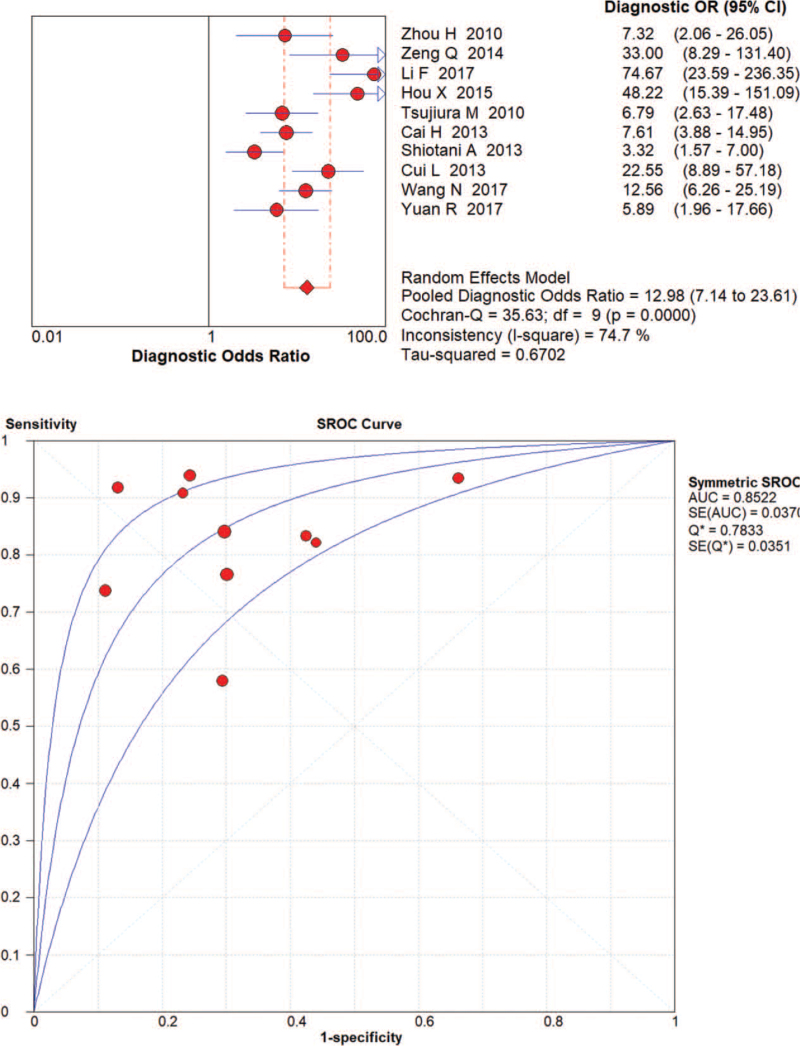
The DOR, AUC, and Q value of miR-106 in the diagnosis of GC.

**Figure 5:**
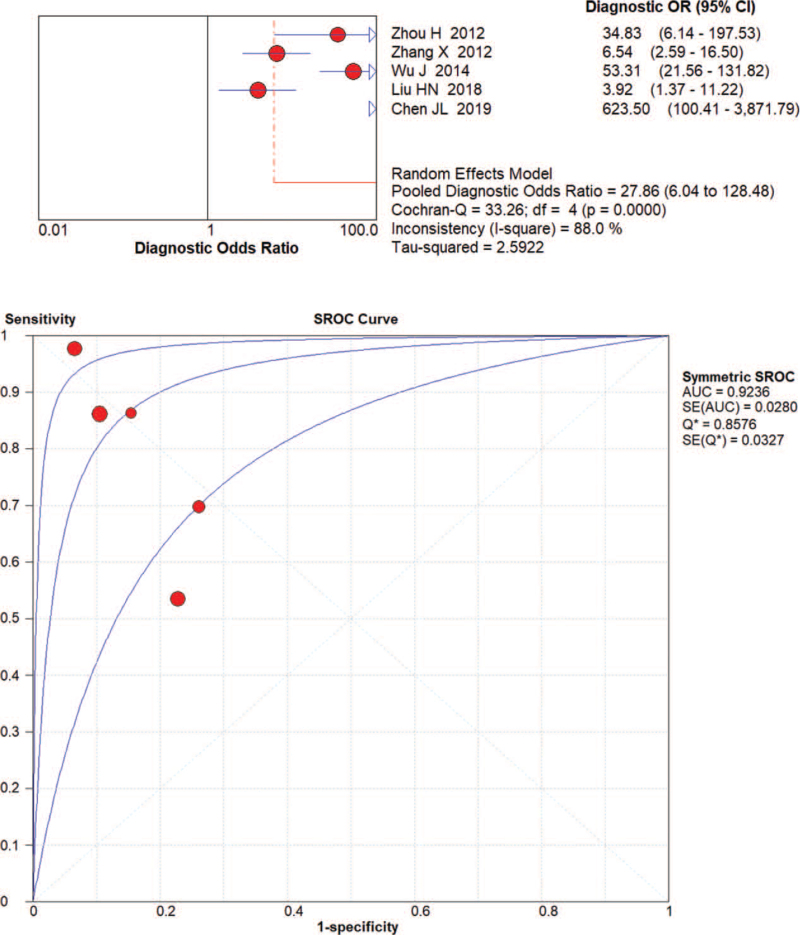
The DOR, AUC, and Q value of miR-421 in the diagnosis of GC.

**Figure 6:**
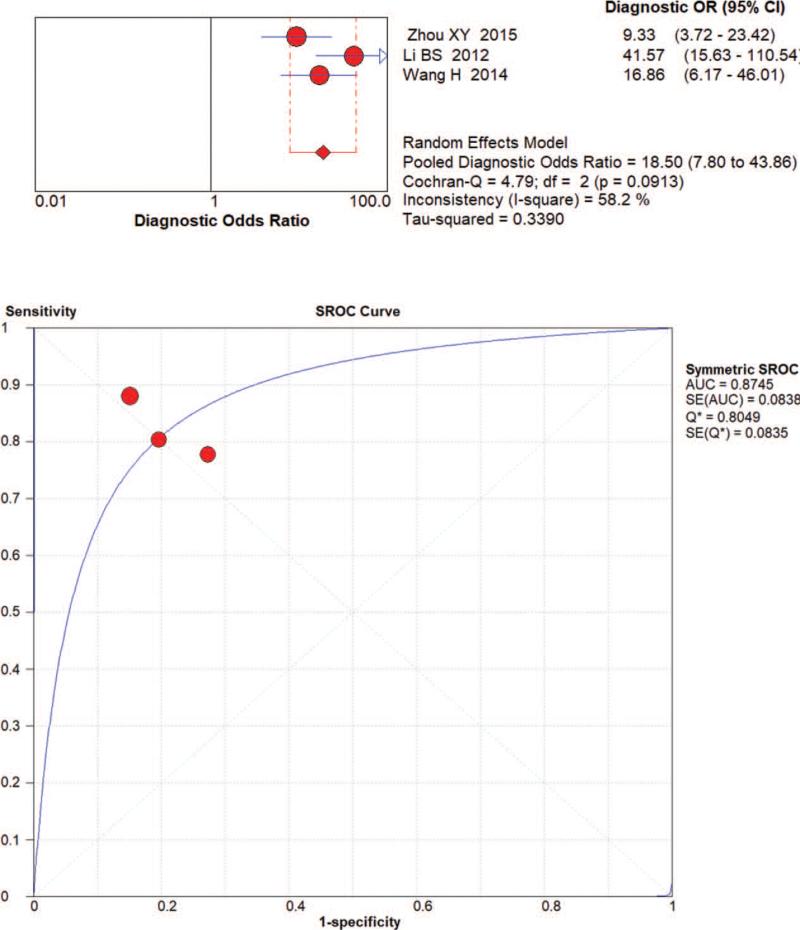
The DOR, AUC, and Q value of miR-223 in the diagnosis of GC.

The random effects model was applied to evaluate the pooled analysis. The DOR, AUC and Q value of miRNA-21 were 12.37 (95% confidence interval [CI]: 5.36–28.54), 0.86 and 0.79, respectively (Fig. 3). The DOR, AUC and Q value of miRNA-106 were 12.98 [95% CI: 7.14–23.61], 0.85 and 0.78, respectively (Fig. 4). The DOR, AUC and Q value of miRNA-421 were 27.86 [95% CI: 6.04–128.48], 0.92 and 0.86, respectively (Fig. 5). The DOR, AUC and Q value of miRNA-223 were 18.50 [95% CI: 7.80–43.86], 0.87 and 0.80, respectively (Fig. 6). These results indicate that miRNA-421 has the highest diagnostic accuracy, followed by miR-223, miRNA-21 and miRNA-106 among the 4 microRNAs in GC.

## 4 Discussion

Early GC is easy to be ignored or mistaken for stomach disease since there are no obvious specific symptoms. However, early detection of GC is pivotal to improve the survival rate and prognosis of GC. Although endoscopy is a highly reliable method for the diagnosis of GC, it is unlikely to be widely used, especially in developing countries, due to the financial burden and fear of physical discomfort caused by endoscopy.^[51]^ To date, the widely used approach for early detection of GC is a number of serum biomarkers, such as cancer embryo antigen, carbohydrate antigen 199, and carbohydrate antigen 724, but their sensitivity and specificity are very low.^[7]^ Thus, a novel effective serum biomarker is urgently needed.

In recent years, aberrantly expressed microRNAs have gained widespread attention as potential biomarkers for early detection of GC.^[52]^ Firstly, microRNAs have relatively high stability and specificity in substantial post-transcriptional regulation and expression. Second, microRNAs are stable in tissues, cells and peripheral blood because they are short and resistant to degradation.^[53]^ Third, each microRNA is specifically expressed in tissue specimens and microRNAs have been shown to be differentially expressed in GC vs. normal tissues. Finally, altered expression of microRNAs in GC is involved in the tumorigenesis and cancer development.

Increasing evidences reported that miR-21, miR-106, miR-421, miR-34, miR-17, miR-25, and miR-133b are dysregulated in GC and could be used as potential diagnostic biomarkers.^[31,41,48,54,55]^ However, previous studies have the defects of a small number of included studies, inconsistent results and few types of microRNA. In GC cells, down-regulation of miR-21 inhibits cell proliferation and EMT, thereby inhibiting invasion and metastasis of tumor cells,^[56,57]^ while miR-106 has similar biological effects on GC, colorectal cancer and endometrial cancer cells.^[58–60]^ MiR-421 and miR-223 regulates the apoptosis and invasion ability of GC cells by targeting Caspase-3 and Arid1a respectively.^[61,62]^ These 4 miRNAs are also involved in regulating drug resistance of GC cells. MiR-21 regulates cisplatin resistance of GC cells through the PI3K/Akt/mTOR pathway.^[63]^ MiR-421 was involved in regulating 5-fluorouracil and gemcitabine resistance in MGC-803 GC cell lines and pancreatic cancer cell lines.^[64,65]^ The sensitivity of GC cells to cisplatin and trastuzumab was regulated by miR-223/FBXW7 axis.^[66,67]^ Therefore, the purpose of this study was to compare the diagnostic value of these 4 miRNAs in GC.

In this study, we retrieved a total of 22 published articles reporting the diagnostic value of miR-21 (n = 9), miR-106 (n = 10), miR-421 (n = 5) and miR-223 (n = 3) in GC. The ROC analysis revealed the AUC value was 0.86 for miR-21, 0.85 for miR-106, 0.92 for miR-421 and 0.87 for miR-223. Our data supported that miRNA-421 has the highest diagnostic accuracy, followed by miR-223, miRNA-21 and miRNA-106 among the 4 microRNAs in GC.

Nevertheless, substantial heterogeneity existed in this study. That may cause by different types of samples (plasma, serum, gastric juice, cells), different portion of early stage, different source of samples and limited number of included studies. Another disadvantage of this study is the included studies mainly from China or Japan, indicating that publication bias existed. Therefore, future large-size study is needed to validate our finding.

In conclusion, despite the limitations mentioned above, the current evidence suggests that miR-21, miR-106, miR-421, and miR-223 have good diagnostic efficacy, especially miR-421, could assist in early diagnosis and mass screening of GC as a noninvasive indicator.

## Author contributions

**Conceptualization:** Liang Zong.

**Data curation:** Yingying Xu, Guiping Wang.

**Formal analysis:** Guiping Wang.

**Funding acquisition:** Liang Zong.

**Investigation:** Wenqing Hu, Ping Chen.

**Methodology:** Songbing He.

**Supervision:** Yongshun Gao, Duonan Yu.

**Validation:** Jinjie Zhang, Ping Chen.

**Visualization:** Guiping Wang.

**Writing – original draft:** Yingying Xu.

**Writing – review & editing:** Dandan Li, Jinjie Zhang.

## References

[1] Sung H Ferlay J Siegel RL. Global cancer statistics 2020: GLOBOCAN estimates of incidence and mortality worldwide for 36 cancers in 185 countries. CA Cancer J Clin 2021;71:209–49. 33538338 10.3322/caac.21660

[2] Torre LA Bray F Siegel RL Ferlay J Lortet-Tieulent J Jemal A. Global cancer statistics. CA Cancer J Clin 2012;65:87–108. 10.3322/caac.21262 25651787

[3] Information Committee of Korean Gastric Cancer A,. Korean gastric cancer association nationwide survey on gastric cancer in 2014. J Gastric Cancer 2016;16:131–40. 27752390 10.5230/jgc.2016.16.3.131 PMC5065942

[4] Katai H Ishikawa T Akazawa K. Five-year survival analysis of surgically resected gastric cancer cases in Japan: a retrospective analysis of more than 100,000 patients from the nationwide registry of the Japanese Gastric Cancer Association (2001-2007). Gastric Cancer 2018;21:144–54. 28417260 10.1007/s10120-017-0716-7

[5] Takahashi T Saikawa Y Kitagawa Y. Gastric cancer: current status of diagnosis and treatment. Cancers (Basel) 2013;5:48–63. 24216698 10.3390/cancers5010048 PMC3730304

[6] CZ H KH Z Q L XH L Y H NH L. Combined use of AFP, CEA, CA125 and CAl9-9 improves the sensitivity for the diagnosis of gastric cancer. BMC Gastroenterol 2013;13:87. 23672279 10.1186/1471-230X-13-87 PMC3655895

[7] Carthew RW Sontheimer EJ. Origins and mechanisms of miRNAs and siRNAs. Cell 2009;136:642–55. 19239886 10.1016/j.cell.2009.01.035 PMC2675692

[8] Acunzo M Romano G Wernicke D Croce CM. MicroRNA and cancer--a brief overview. Adv Biol Regul 2015;57:01–9. 10.1016/j.jbior.2014.09.013 25294678

[9] Di Leva G Garofalo M Croce CM. MicroRNAs in cancer. Annu Rev Pathol 2014;9:287–314. 24079833 10.1146/annurev-pathol-012513-104715 PMC4009396

[10] Lin S Gregory RI. MicroRNA biogenesis pathways in cancer. Nat Rev Cancer 2015;15:321–33. 25998712 10.1038/nrc3932 PMC4859809

[11] Zaheer U Faheem M Qadri I. Expression profile of MicroRNA: an emerging hallmark of cancer. Curr Pharm Des 2019;25:642–53. 30914015 10.2174/1386207322666190325122821

[12] Bica-Pop C Cojocneanu-Petric R Magdo L Raduly L Gulei D Berindan-Neagoe I. Overview upon miR-21 in lung cancer: focus on NSCLC. Cell Mol Life Sci 2018;75:3539–51. 30030592 10.1007/s00018-018-2877-x PMC11105782

[13] Cao J Zhang Y Mu J Yang D Gu X Zhang J. Exosomal miR-21-5p contributes to ovarian cancer progression by regulating CDK6. Hum Cell 2021;34:1185–96. 33813728 10.1007/s13577-021-00522-2

[14] Guan E Liu H Xu N. Lidocaine suppresses gastric cancer development through Circ_ANO5/miR-21-5p/LIFR Axis. Dig Dis Sci 2021. 10.1007/s10620-021-07055-6 34050852

[15] Jiang R Chen X Ge S. MiR-21-5p induces pyroptosis in colorectal cancer via TGFBI. Front Oncol 2021;10:610545. 33614494 10.3389/fonc.2020.610545 PMC7892456

[16] Li Q Li B Li Q. Exosomal miR-21-5p derived from gastric cancer promotes peritoneal metastasis via mesothelial-to-mesenchymal transition. Cell Death Dis 2018;9:854. 30154401 10.1038/s41419-018-0928-8 PMC6113299

[17] Soeda N Iinuma H Suzuki Y. Plasma exosome-encapsulated microRNA-21 and microRNA-92a are promising biomarkers for the prediction of peritoneal recurrence in patients with gastric cancer. Oncol Lett 2019;18:4467–80. 31611956 10.3892/ol.2019.10807 PMC6781766

[18] Peng Q Shen Y Lin K Zou L Shen Y Zhu Y. Comprehensive and integrative analysis identifies microRNA-106 as a novel non-invasive biomarker for detection of gastric cancer. J Transl Med 2018;16:127. 29764446 10.1186/s12967-018-1510-y PMC5952699

[19] Jiang Z Guo J Xiao B. Increased expression of miR-421 in human gastric carcinoma and its clinical association. J Gastroenterol 2010;45:17–23. 19802518 10.1007/s00535-009-0135-6

[20] Lin XF Zhang CQ Dong BR. MiR-421 expression independently predicts unfavorable overall survival in patients with esophageal adenocarcinoma. Eur Rev Med Pharmacol Sci 2019;23:3790–8. 31115005 10.26355/eurrev_201905_17805

[21] Xue L Yang D. MiR-421 inhibited proliferation and metastasis of colorectal cancer by targeting MTA1. J BUON 2018;23:1633–9. 30610787

[22] Zhang Y Gong W Dai S. Downregulation of human farnesoid X receptor by miR-421 promotes proliferation and migration of hepatocellular carcinoma cells. Mol Cancer Res 2012;10:516–22. 22446874 10.1158/1541-7786.MCR-11-0473

[23] Zhong XY Yu JH Zhang WG. MicroRNA-421 functions as an oncogenic miRNA in biliary tract cancer through down-regulating farnesoid X receptor expression. Gene 2012;493:44–51. 22146319 10.1016/j.gene.2011.11.028

[24] Gao Y Lin L Li T Yang J Wei Y. The role of miRNA-223 in cancer: Function, diagnosis and therapy. Gene 2017;616:01–7. 10.1016/j.gene.2017.03.021 28322994

[25] Li J Guo Y Liang X. MicroRNA-223 functions as an oncogene in human gastric cancer by targeting FBXW7/hCdc4. J Cancer Res Clin Oncol 2012;138:763–74. 22270966 10.1007/s00432-012-1154-x PMC11824240

[26] Hu Y Yi B Chen X Xu L Zhou X Zhu X. MiR-223 promotes tumor progression via targeting RhoB in gastric cancer. J Oncol 2022;2022:6708871. 35035482 10.1155/2022/6708871 PMC8758265

[27] Kang W Tong JH Chan AW. Stathmin1 plays oncogenic role and is a target of microRNA-223 in gastric cancer. PLoS One 2012;7:e33919. 22470493 10.1371/journal.pone.0033919 PMC3314670

[28] Gao H Ma J Cheng Y Zheng P. Exosomal transfer of macrophage-derived miR-223 confers doxorubicin resistance in gastric cancer. Onco Targets Ther 2020;13:12169–79. 33268995 10.2147/OTT.S283542 PMC7701146

[29] Cui L Zhang X Ye G. Gastric juice MicroRNAs as potential biomarkers for the screening of gastric cancer. Cancer 2013;119:1618–26. 23335180 10.1002/cncr.27903

[30] Li BS Zhao YL Guo G. Plasma microRNAs, miR-223, miR-21 and miR-218, as novel potential biomarkers for gastric cancer detection. PLoS One 2012;7:e41629. 22860003 10.1371/journal.pone.0041629 PMC3408505

[31] Wu J Li G Wang Z. Circulating MicroRNA-21 is a potential diagnostic biomarker in gastric cancer. Dis Markers 2015;2015:435656. 26063956 10.1155/2015/435656 PMC4433679

[32] Liu HN Wu H Tseng YJ. Serum microRNA signatures and metabolomics have high diagnostic value in gastric cancer. BMC Cancer 2018;18:415. 29653559 10.1186/s12885-018-4343-4 PMC5899358

[33] Zheng Y Cui L Sun W. MicroRNA-21 is a new marker of circulating tumor cells in gastric cancer patients. Cancer Biomark 2011;10:71–7. 22430134 10.3233/CBM-2011-0231 PMC13016254

[34] Wang B Zhang Q. The expression and clinical significance of circulating microRNA-21 in serum of five solid tumors. J Cancer Res Clin Oncol 2012;138:1659–66. 22638884 10.1007/s00432-012-1244-9 PMC11824721

[35] Shiotani A Murao T Kimura Y. Identification of serum miRNAs as novel non-invasive biomarkers for detection of high risk for early gastric cancer. Br J Cancer 2013;109:2323–30. 24104965 10.1038/bjc.2013.596 PMC3817334

[36] Tsujiura M Ichikawa D Komatsu S. Circulating microRNAs in plasma of patients with gastric cancers. Br J Cancer 2010;102:1174–9. 20234369 10.1038/sj.bjc.6605608 PMC2853097

[37] J S C F B H. Application ofmiR-21 and let-7a in serumfor non-invasive diagnosis of gastric cancer and evaluation of surgery results. J Zhengzhou Univers 2012;47:722–5.

[38] Zhou H Guo JM Lou YR. Detection of circulating tumor cells in peripheral blood from patients with gastric cancer using microRNA as a marker. J Mol Med (Berl) 2010;88:709–17. 20349219 10.1007/s00109-010-0617-2

[39] Zeng Q Jin C Chen W. Downregulation of serum miR-17 and miR-106b levels in gastric cancer and benign gastric diseases. Chin J Cancer Res 2014;26:711–6. 25561770 10.3978/j.issn.1000-9604.2014.12.03 PMC4279196

[40] Li F Guo Y Liu J Zhang R. The significance of elevated plasma expression of microRNA 106b∼25 clusters in gastric cancer. PLoS One 2017;12:e0178427. 28562634 10.1371/journal.pone.0178427 PMC5451054

[41] X H M Z HQ Q. Diagnostic significance of miR-106a in gastric cancer. Int J Clin Exp Pathol 2015;8:13096–101. 26722506 PMC4680451

[42] Cai H Yuan Y Hao YF Guo TK Wei X Zhang YM. Plasma microRNAs serve as novel potential biomarkers for early detection of gastric cancer. Med Oncol 2013;30:452. 23307259 10.1007/s12032-012-0452-0

[43] Wang N Wang L Yang Y Gong L Xiao B Liu X. A serum exosomal microRNA panel as a potential biomarker test for gastric cancer. Biochem Biophys Res Commun 2017;493:1322–8. 28986250 10.1016/j.bbrc.2017.10.003

[44] R Y G W Z X H Z H C Y H. Up-regulated circulating miR-106a by DNA methylation promised a potential diagnostic and prognostic marker for gastric cancer. Anticancer Agents Med Chem 2016;16:1093–100. 26179261 10.2174/1871520615666150716110657

[45] Zhou H Xiao B Zhou F. MiR-421 is a functional marker of circulating tumor cells in gastric cancer patients. Biomarkers 2012;17:104–10. 22263628 10.3109/1354750X.2011.614961

[46] Zhang X Cui L Ye G. Gastric juice microRNA-421 is a new biomarker for screening gastric cancer. Tumour Biol 2012;33:2349–55. 22926798 10.1007/s13277-012-0497-x

[47] Wu J Li G Yao Y Wang Z Sun W Wang J. MicroRNA-421 is a new potential diagnosis biomarker with higher sensitivity and specificity than carcinoembryonic antigen and cancer antigen 125 in gastric cancer. Biomarkers 2015;20:58–63. 25510566 10.3109/1354750X.2014.992812

[48] Chen J Wu L Sun Y. Mir-421 in plasma as a potential diagnostic biomarker for precancerous gastric lesions and early gastric cancer. PeerJ 2019;7:e7002. 31245174 10.7717/peerj.7002 PMC6585904

[49] XY Z GP J H C WJ J CQ Y GX Z. Clinical role of circulating miR-223 as a novel biomarker in early diagnosis of cancer patients. Int J Clin Exp Med 2015;8:16890–8. 26629240 PMC4659128

[50] Wang H Wang L Wu Z. Three dysregulated microRNAs in serum as novel biomarkers for gastric cancer screening. Med Oncol 2014;31:298. 25367852 10.1007/s12032-014-0298-8

[51] Zong L Abe M Seto Y Ji J. The challenge of screening for early gastric cancer in China. Lancet 2016;388:2606. 27894662 10.1016/S0140-6736(16)32226-7

[52] H I M K H T. Role of microRNAs in gastric cancer. World J Gastroenterol 2014;20:5694–9. 24914330 10.3748/wjg.v20.i19.5694 PMC4024779

[53] Chen X Ba Y Ma L. Characterization of microRNAs in serum: a novel class of biomarkers for diagnosis of cancer and other diseases. Cell Res 2008;18:997–1006. 18766170 10.1038/cr.2008.282

[54] Jafari N Abediankenari S. MicroRNA-34 dysregulation in gastric cancer and gastric cancer stem cell. Tumour Biol 2017;39:1010428317701652. 28468587 10.1177/1010428317701652

[55] ZiaSarabi P Sorayayi S Hesari A Ghasemi F. Circulating microRNA-133, microRNA-17 and microRNA-25 in serum and its potential diagnostic value in gastric cancer. J Cell Biochem 2019;120:12376–81. 30861177 10.1002/jcb.28503

[56] Quan J Dong D Lun Y. Circular RNA circHIAT1 inhibits proliferation and epithelial-mesenchymal transition of gastric cancer cell lines through downregulation of miR-21. J Biochem Mol Toxicol 2020;34:e22458. 32020707 10.1002/jbt.22458

[57] Xiao T Jie Z. MiR-21 promotes the invasion and metastasis of gastric cancer cells by activating epithelial-mesenchymal transition. Eur Surg Res 2019;60:208–18. 31722341 10.1159/000504133

[58] Li X Yi X Bie C Wang Z. Expression of miR-106 in endometrial carcinoma RL95-2 cells and effect on proliferation and invasion of cancer cells. Oncol Lett 2018;16:2251–4. 30008926 10.3892/ol.2018.8926 PMC6036432

[59] Zhang GJ Li JS Zhou H Xiao HX Li Y Zhou T. MicroRNA-106b promotes colorectal cancer cell migration and invasion by directly targeting DLC1. J Exp Clin Cancer Res 2015;34:73. 26223867 10.1186/s13046-015-0189-7 PMC4520100

[60] Zhu Z Yang Q Zhang B Wu W Yuan F Zhu Z. miR-106b promotes metastasis of early gastric cancer by targeting ALEX1 in vitro and in vivo. Cell Physiol Biochem 2019;52:606–16. 30907988 10.33594/000000043

[61] Wu JH Yao YL Gu T. MiR-421 regulates apoptosis of BGC-823 gastric cancer cells by targeting caspase-3. Asian Pac J Cancer Prev 2014;15:5463–8. 25041019 10.7314/apjcp.2014.15.13.5463

[62] Zhu Y Li K Yan L He Y Wang L Sheng L. miR-223-3p promotes cell proliferation and invasion by targeting Arid1a in gastric cancer. Acta Biochim Biophys Sin (Shanghai) 2020;52:150–9. 31912865 10.1093/abbs/gmz151

[63] Gu Y Fei Z Zhu R. miR-21 modulates cisplatin resistance of gastric cancer cells by inhibiting autophagy via the PI3K/Akt/mTOR pathway. Anticancer Drugs 2020;31:385–93. 31913198 10.1097/CAD.0000000000000886

[64] Jingyue S Xiao W Juanmin Z Wei L Daoming L Hong X. TFAP2E methylation promotes 5fluorouracil resistance via exosomal miR106a5p and miR421 in gastric cancer MGC803 cells. Mol Med Rep 2019;20:323–31. 31115533 10.3892/mmr.2019.10237 PMC6579997

[65] Shopit A Li X Wang S. Enhancement of gemcitabine efficacy by K73-03 via epigenetically regulation of miR-421/SPINK1 in gemcitabine resistant pancreatic cancer cells. Phytomedicine 2021;91:153711. 34450377 10.1016/j.phymed.2021.153711

[66] Eto K Iwatsuki M Watanabe M. The sensitivity of gastric cancer to trastuzumab is regulated by the miR-223/FBXW7 pathway. Int J Cancer 2015;136:1537–45. 25159729 10.1002/ijc.29168

[67] Zhou X Jin W Jia H Yan J Zhang G. MiR-223 promotes the cisplatin resistance of human gastric cancer cells via regulating cell cycle by targeting FBXW7. J Exp Clin Cancer Res 2015;34:28. 25888377 10.1186/s13046-015-0145-6 PMC4387683

